# *Arabidopsis thaliana* Cyclic Nucleotide‐Gated Channel 19 is involved in root extracellular ATP and Pep1 signalling

**DOI:** 10.1111/nph.70624

**Published:** 2025-10-05

**Authors:** Youzheng Ning, Bryony C. I. C. Jacobs, Clementine Langlet, Limin Wang, Zhizhong Song, Adeeba M. Dark, Elsa Matthus, Sebastian Eves‐van den Akker, Taufiq Rahman, Julia M. Davies

**Affiliations:** ^1^ Department of Plant Sciences University of Cambridge Cambridge CB2 3EA UK; ^2^ Département Génie Biologique Polytech Clermont Campus Universitaire des Cézeaux, 2 Ave Blaise Pascal 63 178 Aubière France; ^3^ Department of Plant and Microbial Biology University of Zurich Zollikerstrasse 107 8008 Zurich Switzerland; ^4^ College of Horticulture Ludong University 186 Hongqizhong Road Yantai 264025 China; ^5^ Leibniz Centre for Agricultural Landscape Research (ZALF e.V.) Eberswalder Straße 84 15374 Müncheberg Germany; ^6^ Crop Science Centre Lawrence Weaver Road Cambridge CB3 0LE UK; ^7^ Department of Pharmacology University of Cambridge Tennis Court Road Cambridge CB2 1QR UK

**Keywords:** Arabidopsis, ATP, calcium, channel, CNGC19, DAMP, PEPR, root

## Disclaimer

The New Phytologist Foundation remains neutral with regard to jurisdictional claims in maps and in any institutional affiliations.

## Introduction

Extracellular ATP (eATP) can function as a damage‐associated molecular pattern (DAMP) that signals wounding incurred by microbial ingress and herbivore attacks (Tanaka & Heil, 2021; Yuan *et al*., 2025). *Arabidopsis thaliana* root and leaf wounding trigger rapid accumulation of eATP (Dark *et al*., 2011; Myers *et al*., 2022). eATP is then sensed by the plasma membrane (PM) co‐receptors Does Not Respond to Nucleotide1/P2‐type purinoceptor Kinase 1 (DORN1/P2K1) and P2K2 (Choi *et al*., 2014; Pham *et al*., 2020). The perception of eATP leads to downstream signalling cascades involving elevation of cytosolic free calcium ([Ca^2+^]_cyt_), reactive oxygen species and nitric oxide plus MAP (Mitogen‐Activated Protein) kinase activation (Demidchik *et al*., 2003; Foresi *et al*., 2007; Chen *et al*., 2017; Kim *et al*., 2023). Analyses of eATP‐induced transcriptomes point to DORN1/P2K1‐dependent regulation of multiple defence‐related hormone signalling pathways involving jasmonic acid (JA), ethylene and salicylic acid (Tripathi *et al*., 2017; Jewell *et al*., 2019). Accordingly, loss‐of‐function DORN1/P2K1 and P2K2 mutants show increased susceptibility to a variety of plant pathogens, including fungi (*Rhizoctonia solani* and *Sclerotinia sclerotiorum*), oomycetes (*Plasmodiophora brassicae*, *Phytophthora infestans* and *Phytophthora capsici*) and bacteria (*Pseudomonas syringae*) plus insect herbivores (*Pieris rapae*, *Salix exigua* and *Spodoptera litura*), whilst receptor overexpression lines reduce plant susceptibility (Bouwmeester *et al*., 2014; Balagué *et al*., 2017; Chen *et al*., 2017; Jewell *et al*., 2022; Kumar *et al*., 2020; Kundu *et al*., 2025; Yuan *et al*., 2025).

[Ca^2+^]_cyt_ serves as an important second messenger in plant stress and immunity signalling (Dong *et al*., 2022; Köster *et al*., 2022). eATP induces a specific biphasic [Ca^2+^]_cyt_ ‘signature’ in roots through DORN1/P2K1, with the possibility of a relatively minor involvement of P2K2 or as yet unknown receptors (Demidchik *et al*., 2003; Zhu *et al*., 2018, 2020; Matthus *et al*., 2019a,b, 2022; Smith *et al*., 2021). How eATP receptors initiate this [Ca^2+^]_cyt_ increase in roots is not fully understood (Sun *et al*., 2021), but the PM Cyclic Nucleotide‐Gated Channel2 (CNGC2) is involved, with the possibility of CNGC4 and CNGC6 also (Duong *et al*., 2022; Wang *et al*., 2022). The *Arabidopsis* CNGC family consists of 20 channel‐subunit members, with important functions in signalling (Jarratt‐Barnham *et al*., 2021). CNGC2 has recently been shown to be phosphorylated by DORN1/P2K1 in leaves (Sun *et al*., 2025) and was reported to mediate the *Arabidopsis* root's [Ca^2+^]_cyt_ response to eATP (Wang *et al*., 2022). However, the significant residual [Ca^2+^]_cyt_ increase in the loss‐of‐function *cngc2* mutant indicates that other channels also support the eATP‐induced [Ca^2+^]_cyt_ signal in roots (Wang *et al*., 2022). Studies have revealed Cyclic Nucleotide‐Gated Channel 19 (CNGC19) involvement as a PM Ca^2+^‐permeable channel in generating the [Ca^2+^]_cyt_ signal and defence signalling pathways induced by herbivore attack on leaves and fungal colonisation of roots (Meena *et al*., 2019; Jogawat *et al*., 2020). Both of these challenges can trigger eATP accumulation through wounding (Nizam *et al*., 2019; Myers *et al*., 2022). Moreover, *CNGC19* and *DORN1/P2K1* are co‐expressed whilst eATP upregulates DORN1/P2K1‐dependent *CNGC19* expression specifically in roots (Tripathi *et al*., 2017; Jewell *et al*., 2019; Sowders & Tanaka, 2023). Such findings suggest that CNGC19 may be part of the eATP response pathway. Indeed, a recent study by Kundu *et al*. (2025) has shown that CNGC19 is part of the whole seedling [Ca^2+^]_cyt_ response to eATP (measured using cytosolic aequorin in the *cngc19‐2* loss‐of‐function mutant) and that CNGC19 is a possible interacting partner of DORN1/P2K1 (Kundu *et al*., 2025). This begs the question of whether CNGC19 operates in the root's response to eATP.

In this study, CNGC19 was found to make a significant contribution to the *Arabidopsis* root's eATP‐induced [Ca^2+^]_cyt_ increase (determined using the intensiometric GCaMP3 [Ca^2+^]_cyt_ reporter (Vincent *et al*., 2017) in the *cngc19‐1* T‐DNA insertion loss‐of‐function mutant). The root transcriptional response to eATP revealed amplification of *CNGC19* expression through DORN1/P2K1, but only partially through CNGC2. Transcription of specific eATP‐induced defence‐related genes was found to require CNGC19, indicative of discrete signalling pathways. These genes included those for the DAMP PM Plant Endogenous Peptide Receptors *PEPR1* and *PEPR2*. As CNGC19 is also needed for Pep1‐induced [Ca^2+^]_cyt_ increase (determined with *cngc19‐2* mutant seedlings by Meena *et al*., 2019 and also here with *cngc19‐1* roots), together the results point to CNGC19 as a common component of these two DAMP pathways.

## Results and Discussion

### CNGC19 contributes to eATP‐induced [Ca^2+^]_cyt_ increase in roots

**Fig. 1:**
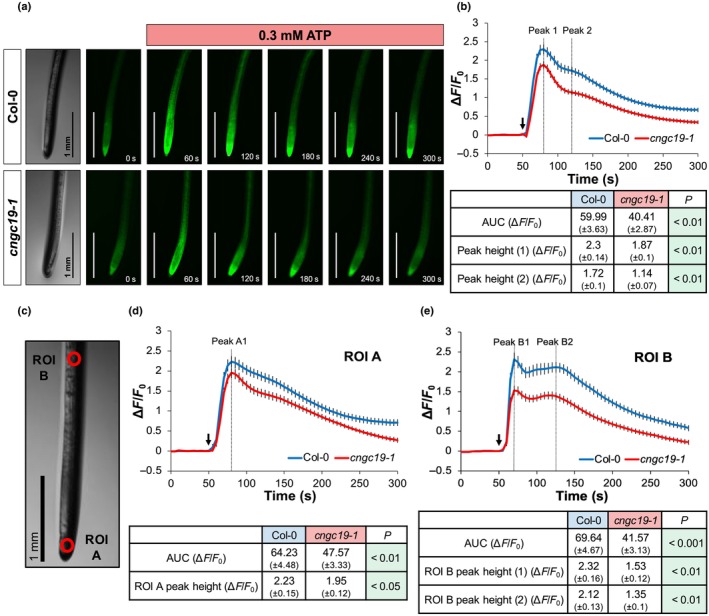
Root extracellular ATP (eATP)‐induced cytosolic free calcium ([Ca^2+^]_cyt_) response is significantly reduced in *Arabidopsis thaliana cyclic nucleotide‐gated channel19‐1* (*cngc19‐1*). eATP‐induced fluorescence (*F*) was measured for 250 s in Columbia‐0 (Col‐0) and *cngc19‐1* roots (expressing cytosolic GCaMP3) following a 0.3 mM eATP treatment at 50 s. (a) Intensiometric images (including a brightfield) taken from a single representative Col‐0 or *cngc19‐1* root across a specified time series. The 0.3 mM eATP treatment is indicated by a box. Bars, 1 mm. (b) Mean ± SE normalised GCaMP3 fluorescence (Δ*F*/*F*_0_) in the full root tip (*c*. 2.5 mm) in response to 0.3 mM eATP in Col‐0 and *cngc19‐1*. The black arrow indicates application of a 1 μl 0.3 mM eATP treatment (50 s). Two Δ*F*/*F*_0_ peaks (Peak 1, 80 s; Peak 2, 120 s) are shown by dashed lines. eATP‐dependent area under the curve (AUC) and peak height analyses are also shown (±SE), with significant differences (*P*) between Col‐0 and *cngc19‐1* determined by Student's *t*‐test. (c) Brightfield image of a root tip annotated with the two regions of interest (ROI) investigated, ‘A’ and ‘B’. ROI A represents a 150 μm^2^ region found within the first 1 mm of the root apex and ROI B represents a 150 μm^2^ region *c*. 2.5 mm away from the root apex. Mean ± SE normalised GCaMP3 fluorescence (Δ*F*/*F*_0_) measured in response to 0.3 mM eATP in (d) ROI A and (e) ROI B in Col‐0 and *cngc19‐1*. eATP‐induced AUC and Δ*F*/*F*_0_ peak height analyses are also included; (d) ROI A (Peak A1, 80 s) and (e) ROI B (Peak B1, 70 s; Peak B2, 125 s). The significance value (*P*) for differences between Col‐0 and *cngc19‐1* were determined by a Student's *t‐*test. Data were obtained across six biological replicates with *n* = 39 (Col‐0) and *n* = 35 (*cngc19‐1*).

Columbia‐0 (Col‐0) and *cngc19‐1* (both expressing cytosolic 35S::GCaMP3; Supporting Information Fig. S1a) were used to test for CNGC19's involvement in the root's biphasic [Ca^2+^]_cyt_ response to 0.3 mM eATP (the same concentration used in previous work on CNGC2; Wang *et al*., 2022) (Methods S1–S3; Fig. 1a; Videos S1, S2). Analysis of the eATP‐induced [Ca^2+^]_cyt_ increase (Fig. 1b) showed that the *cngc19‐1* mutant was significantly impaired in the magnitude of the biphasic maximal responses (‘peaks’) and total [Ca^2+^]_cyt_ mobilised (estimated as area under the curve (AUC)). Previous research using GCaMP3 and aequorin has shown that the root apex generates the first increment (Peak 1) of the [Ca^2+^]_cyt_ increase and the semi‐autonomous Peak 2 then occurs sub‐apically (Matthus *et al*., 2019a,b, 2022). Here, a region of interest (ROI) was placed at the apex (ROI A) and sub‐apically (ROI B; Fig. 1c). Rapid and distinctive fluorescence increases occurred at both ROI A and ROI B in response to 0.3 mM eATP, with *cngc19‐1* showing significantly lower maximum responses and AUC in both ROIs compared with Col‐0 (Fig. 1d,e). There were no significant differences between genotypes in response to 0.6 mM NaCl control solution (Fig. S1b–f; Videos S3, S4) whilst the eATP‐induced [Ca^2+^]_cyt_ response was significantly greater than the control in both genotypes (Fig. S2). With normal expression of the *DORN1/P2K1* and *P2K2* eATP receptors evident in *cngc19‐1* roots (Fig. S3a,b, measured by quantitative reverse transcription polymerase chain reaction (qRT‐PCR); Methods S3, S4; Table S1), the lesions in the mutant's [Ca^2+^]_cyt_ response to eATP are unlikely to be the result of impaired ability to sense eATP and instead point to CNGC19's acting as a PM Ca^2+^ channel in eATP signalling.

### *CNGC19* expression is DORN1/P2K1‐dependent but does not affect receptor expression

eATP's inability to increase eATP receptor expression agrees with results on roots from a *DORN1/P2K1* GUS reporter line (Sowders & Tanaka, 2023). By contrast, eATP (0.3 mM) significantly increased *CNGC19* expression in Col‐0 roots in a DORN1/P2K1‐dependent manner; induction was significantly lower in the *dorn1‐3* loss‐of‐function mutant (Fig. S3c,d). Significant induction in Col‐0 was only evident after 30 min, tallying with a previous finding of a slow response to eATP using CNGC19 promoter‐driven GUS expression in roots (Sowders & Tanaka, 2023). Moreover, eATP‐induced *CNGC19* expression was dependent on CNGC2 (which lies downstream of DORN1/P2K1 in root [Ca^2+^] signalling; Wang *et al*., 2022). Significant *CNGC19* induction in Col‐0 and the complemented *cngc2‐3* mutant was evident at 30 min but was significantly impaired in the *cngc2‐3* loss‐of‐function mutant (Fig. S3e). However, a significant level of *CNGC19* upregulation remained in the *cngc2‐3* mutant, suggesting the presence of another pathway downstream of DORN1/P2K1 (Fig. S3e). In contrast to *CNGC19* (but in common with the eATP receptors), *CNGC2* expression was not significantly increased by eATP and was unaffected by loss of CNGC19 function (Fig. S3f). Overall, these results highlight positive feedback in roots between eATP and *CNGC19* expression via the DORN1/P2K1 signalling pathway that is not wholly reliant on CNGC2. They also extend the observation that *DORN1/P2K1* expression in roots is not amplified by eATP (Sowders & Tanaka, 2023) to include *P2K2* and *CNGC2*. Thus, there appear to be modules of eATP signalling components that at the transcriptional level vary in their responsiveness to eATP.

### CNGC19 contributes to eATP‐induced transcriptional responses and may link to immunopeptide DAMP signalling

eATP regulates transcription of immunity‐related genes through DORN1/P2K1 (Tripathi *et al*., 2017; Jewell *et al*., 2019; Yuan *et al*., 2025). Whether this is mediated by CNGC19 has yet to be tested directly in any tissue. Upregulation of *Jasmonic Acid ZIM‐domain 5* (*JAZ5*) by eATP or in *R. solani*‐infected roots is DORN1/P2K1‐dependent (Tripathi *et al*., 2017; Kumar *et al*., 2020). Here, *JAZ5* in roots was significantly upregulated by eATP in Col‐0 but not *cngc19‐1* (Fig. S4a), suggesting that CNGC19 is needed to regulate JA‐mediated responses (Tripathi *et al*., 2017; Jewell *et al*., 2019). Defence‐related genes *WRKY DNA‐Binding Protein 40* and *Mitogen‐Activated Protein Kinase 3*, which are both dependent on DORN1/P2K1 and CNGC2 for eATP upregulation in roots (Wang *et al*., 2022), also required CNGC19 on longer (30 min) eATP exposure (Fig. S4b,c). By contrast, the response of *Calcium‐dependent Protein Kinase 28* (also DORN1/P2K1‐ and CNGC2‐dependent for transcription) encoding a regulatory DORN1/P2K1‐interacting protein (Wang *et al*., 2022; Sowders *et al*., 2024) showed no significant difference between genotypes (Fig. S4d). This suggests a CNGC19‐specific defence‐related transcriptome induced by eATP.

**Fig. 2:**
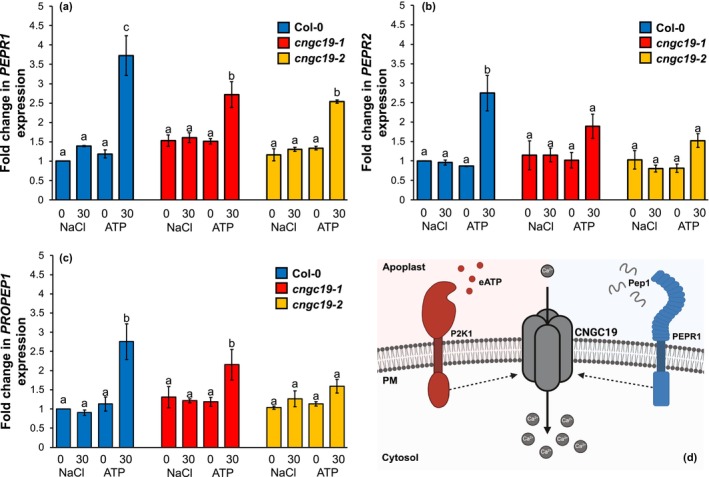
Cyclic Nucleotide‐Gated Channel 19 (CNGC19) is required for extracellular ATP (eATP)‐induced *PEPR1* and *PEPR2* expression in *Arabidopsis thaliana* roots. Whole roots of Col‐0, *cyclic nucleotide gated channel19‐1* (*cngc19‐1*) and *cngc19‐2* were treated with 0.3 mM ATP or 0.6 mM NaCl for 0 or 30 min before RNA extraction was carried out, followed by quantitative reverse transcription polymerase chain reaction (qRT‐PCR). *UBQ10* (*Poly Ubiquitin10*) and *Tubulin beta chain 4* (*TUB4*) were used as housekeeping genes for normalisation. (a) *Plant Endogenous Peptide Receptor1* (*PEPR1*), (b) *PEPR2* and (c) *Pro Peptide1* (*PROPEP1*) expression in Col‐0, *cngc19‐1* and *cngc19‐2*. Data are means (±SE) of three biological replicates, with each biological replicate, including three technical replicates. Different letters indicate significant differences as determined using two‐way ANOVA and a *post hoc* Tukey Honestly Significant Difference (HSD) test. (0, 0 min; 30, 30 min; ATP, 0.3 mM ATP treatment; NaCl, 0.6 mM NaCl treatment). (d) Schematic of the suggested involvement of CNGC19 (shown as a homotetramer for simplicity but heterotetramers are also feasible) in the root's [Ca^2+^]_cyt_ (cytosolic free Ca^2+^) response to eATP and Peptide 1 (Pep1). eATP and Pep1 are recognised by the plasma membrane‐bound receptors, Does Not Respond to Nucleotide1/P2‐type purinoceptor Kinase 1 (DORN1/P2K1) and PEPR1, respectively. Pep1 binding to PEPR2 has been omitted for simplicity. The binding of these putative ligands to their receptors would promote the opening of CNGC19 to mediate the influx of Ca^2+^ to the cytosol. Dashed lines indicate proposed interactions. For eATP, this could include physical interaction (Kundu *et al*., 2025), phosphorylation by the receptor's cytosolic kinase domain or possible production of cyclic guanosine monophosphate (GMP; Sun *et al*., 2021). For the peptide pathway, there is no physical interaction between CNGC19 and PEPR1 (or PEPR2; Meena *et al*., 2019) whilst PEPR1 (and PEPR2) may be capable of cyclic GMP synthesis or phosphorylation by their kinase domains (Qi *et al*., 2010; Ma *et al*., 2012; Liu *et al*., 2013). PM, plasma membrane. Figure created in BioRender (https://BioRender.com/44pdker).

Damaged root cells not only release ATP but also immunopeptides, such as Pep1 (Hander *et al*., 2019). Damage‐induced [Ca^2+^]_cyt_ increase causes activation of the MetaCaspase4 zymogen; this cysteine protease cleaves the inactive precursor PROPEP1 to the active form Pep1, which then exits the cell to activate defence responses by binding to PEPR1 or PEPR2 (Hander *et al*., 2019). eATP upregulates transcription of the *PEPR1*/*PEPR2/PROPEP1* DAMP signalling module through DORN1/P2K1 (Tripathi *et al*., 2017; Jewell *et al*., 2019). Here, eATP upregulation of *PEPR1*/*PEPR2* was impaired in both *cngc19‐1* and *cngc19‐2* roots after 30 min eATP treatment (Fig. 2a,b), although upregulation of PROPEP1 was only significantly impaired in *cngc19‐2* (Fig. 2c). That *cngc19‐1* and *cngc19‐2* do not always appear to be allelic was noted previously by Meena *et al*. (2019). Nevertheless, the *PEPR* data are indicative of a pathway for priming and augmenting longer term DAMP signalling. Indeed, Pep1 upregulates *CNGC19* expression in root meristems (Dhar *et al*., 2025).

That *cngc19‐1* is impaired in eATP‐induced root [Ca^2+^]_cyt_ elevation whilst *cngc19‐2* seedlings are impaired in both eATP‐ and Pep1‐induced [Ca^2+^]_cyt_ increase (Meena *et al*., 2019; Kundu *et al*., 2025) points to CNGC19 as a common component of these two DAMP pathways. Certainly, *CNGC19* co‐expresses with *DORN1/P2K1*, *PEPR1*, *PEPR2* and *PROPEP1* (Obayashi *et al*., 2022). Here, Pep1 induced a [Ca^2+^]_cyt_ increase in roots (measured with GCaMP3) that was significantly impaired in *cngc19‐1* (Videos S5–S8; Fig. S5). Similar basal *PEPR1* and *PEPR2* expression in Col‐0 and *cngc19‐1* roots (Fig. 2a,b) suggests that this impairment is due to loss of channel function. Overall, results point to CNGC19's operating in both eATP and Pep1 pathways in roots. CNGC2 has also been found to function in both eATP and Pep signalling (Qi *et al*., 2010; Wang *et al*., 2022), and it would be interesting to test a *cngc2/cngc19* double mutant. However, such a mutant would be unlikely to yield a complete lack of response to eATP as CNGC4 and CNGC6 are also implicated in that pathway (Duong *et al*., 2022; Wang *et al*., 2022). Whether CNGC19 acts in both eATP and Pep signalling at the level of an individual cell now needs to be determined, as does the scope for utilising this protein in improving plant responses to damage (Fig. 2d).

## Competing interests

None declared.

## Author contributions

YN, BCICJ, LW, ZS, SEA, TR and JMD planned and designed the research. BCICJ, YN, CL, LW, ZS, AMD, EM and JMD performed the experiments and analysed the data. All authors contributed to writing. BCICJ and JMD revised the manuscript. YN and BCICJ contributed equally to this work.

## Supporting information


**Fig. S1** Col‐0 and *cngc19‐1* root [Ca^2+^]_cyt_ response to 0.6 mM NaCl is statistically similar.
**Fig. S2** The eATP‐induced [Ca^2+^]_cyt_ response is significantly larger than the response to control solution in both Col‐0 and *cngc19‐1* roots.
**Fig. S3** eATP receptor expression is independent of CNGC19, but eATP‐induced *CNGC19* expression requires DORN1/P2K1 and CNGC2.
**Fig. S4** CNGC19 is required for eATP‐induced *JAZ5*, *WRKY40* and *MPK3* expression in roots.
**Fig. S5** The root Pep1‐induced [Ca^2+^]_cyt_ response is significantly reduced in *cngc19‐1*.
**Methods S1** Plant materials and growth conditions.
**Methods S2** GCaMP3 fluorescence microscopy.
**Methods S3** Statistical analyses.
**Methods S4** qRT‐PCR gene expression studies.
**Table S1** The primer sequences used in qRT‐PCR.

**Video S1** The eATP‐induced [Ca^2+^]_cyt_ response in a representative Col‐0 root.

**Video S2** The eATP‐induced [Ca^2+^]_cyt_ response in a representative *cngc19‐1* root.

**Video S3** The NaCl‐induced [Ca^2+^]_cyt_ response in a representative Col‐0 root.

**Video S4** The NaCl‐induced [Ca^2+^]_cyt_ response in a representative *cngc19‐1* root.

**Video S5** The Pep1‐induced [Ca^2+^]_cyt_ response in a representative Col‐0 root.

**Video S6** The Pep1‐induced [Ca^2+^]_cyt_ response in a representative *cngc19‐1* root.

**Video S7** The control‐induced [Ca^2+^]_cyt_ response in a representative Col‐0 root.

**Video S8** The control‐induced [Ca^2+^]_cyt_ response in a representative *cngc19‐1* root.
Please note: Wiley is not responsible for the content or functionality of any Supporting Information supplied by the authors. Any queries (other than missing material) should be directed to the *New Phytologist* Central Office.

## Data Availability

All the data and materials that support the findings of this study have been included within the article and in Figs S1–S4.
