# Supplementary material for: Arabidopsis thaliana Cyclic Nucleotide‐Gated Channel 19 is involved in root extracellular ATP and Pep1 signalling
Source: New Phytol. 2025 Oct 5;248(5):2192–7. doi: 10.1111/nph.70624 (PMC12589707; doi:10.1111/nph.70624)
Supplement: Supplementary file 1 — Fig. S1 Col‐0 and cngc19‐1 root [Ca2+]cyt response to 0.6 mM NaCl is statistically similar. Fig. S2 The eATP‐induced [Ca2+]cyt response is significantly larger than the response to control solution in both Col‐0 and cngc19‐1 roots. Fig. S3 eATP receptor expression is independent of CNGC19, but eATP‐induced CNGC19 expression requires DORN1/P2K1 and CNGC2. Fig. S4 CNGC19 is required for eATP‐induced JAZ5, WRKY40 and MPK3 expression in roots. Fig. S5 The root Pep1‐induced [Ca2+]cyt response is significantly reduced in cngc19‐1. Methods S1 Plant materials and growth conditions. Methods S2 GCaMP3 fluorescence microscopy. Methods S3 Statistical analyses. Methods S4 qRT‐PCR gene expression studies. Table S1 The primer sequences used in qRT‐PCR. [file NPH-248-2192-s009.docx]

## *New Phytologist* Supporting Information

Article title: *Arabidopsis thaliana* Cyclic Nucleotide-Gated Channel 19 (CNGC19) is involved in root extracellular ATP and Pep1 signalling

Authors: Youzheng Ning, Bryony C.I.C. Jacobs, Clementine Langlet, Limin Wang, Zhizhong Song, Adeeba M. Dark, Elsa Matthus, Sebastian Eves-van den Akker, Taufiq Rahman, Julia M. Davies

Article acceptance date: 16 September 2025

The following Supporting Information is available for this article:

**Methods S1** **Plant materials and growth conditions.**

All *Arabidopsis thaliana* mutants used were in the Columbia (Col-0) wild type background. *cngc19-1* (SALK_027306) was as described by Kugler et al. (2009). *cngc19-2* (SALK_129200) was obtained from the Nottingham Arabidopsis Stock Centre (NASC). The *dorn1-3* loss of function mutant, the *cngc2-3* loss of function mutant and complementation line (*cngc2-3,CNGC2::CNGC2*) were as described by Wang et al. (2022). Col-0 expressing cytosolic 35S::GCaMP3 was as described by Vincent et al. (2017). The *cngc19-1* 35S::GCaMP3 line was produced by crossing Col-0::GCaMP3 with *cngc19-1*. For seed production, plants were grown in soil under long-day conditions (16 h light/ 8 h dark) in a growth room (200 µmol m^-2^ s^-1^ light intensity, 20 ℃, 60 % relative humidity). Genotyping of the *cngc19-1*::GCaMP3 line was conducted using the following primers: LB: CGCGGATCTCTTTATTCACAC, RB: ATGAGGATTCATTATTCCGGG and LBb1.3: ATTTTGCCGATTTCGGAAC. Surface-sterilised seeds were sown on half-strength Murashige-Skoog (MS; Duchefa Biochemie, Haarlem, The Netherlands) medium, 1 % (w/v) sucrose, 0.8 % (w/v) Bacto-agar (BD Diagnostics VWR, 38800 Le Pont de Claix, France), pH 5.6, and stratified in darkness at 4 ℃ for 48 h. The plates were then transferred into a growth chamber (PERCIVAL, CLF Plant Climatics, Emersacker, Germany) at 21 ℃ with a 16 h light / 8 h dark photoperiod (80 µmol m^-2^ s^-1^) and plants were grown vertically for 9 or 11 days.

**Methods S2** **GCaMP3 fluorescence microscopy.**

The root tips of 9-day-old *cngc19-1* 35S::GCaMP3 seedlings (F_2_ segregating population due to low fluorescence in the F_3_ population) were imaged *in situ* on horizontal growth plates for 300 s, with images collected every 5 s. After 50 s, 1 µL of control (0.6 mM NaCl) or 0.3 mM ATP solution (prepared in half MS liquid medium, pH 5.6, 1 % (w/v) sucrose) was applied directly to the root tip. For Supplementary Figure S5, 1 µL of control (half MS liquid medium, pH 5.6, 1 % (w/v) sucrose) or 1 µM Pep1 solution (prepared in half MS liquid medium, pH 5.6, 1 % (w/v) sucrose) was applied. The Pep1 peptide sequence of ATKVKAKQRGKEKVSSGRPGQHN (Life Science Group) and concentration were those used by Krol et al. (2010). Images were captured using a Stereo microscope M205 FA (Leica) with a DFC365FX camera (Leica) and a Sola SE365 light source (Lumencor) which allowed excitation at 470/40 nm and collected emission at 525/50 nm using an ET-GFP filter (Leica). The camera was set to a 500 ms exposure time, a gain of 2.0 and 30× magnification. For Supplementary Figure S5 the camera was set to a 450 ms exposure time and a gain of 3.0. ImageJ Fiji was used to quantify GCaMP3 fluorescence. The protocols for selecting the regions of interest (ROI) and normalising GCaMP3 fluorescence (ΔF/F_0_) were followed as previously described (Vincent et al., 2017; Matthus et al., 2022). Briefly, ROI A represented a 150 µm^2^ region found at the apical root and ROI B represented a 150 µm^2^ region located in the sub-apex, ~2.5 mm away from the root tip. After imaging, each individual seedling was genotyped to confirm its genetic background with only homozygous *19-1* seedlings used for the data presented in this work.

**Methods S3 Statistical analyses.**

All data collected were analysed using Excel 2019 and R programme (https://www.r-project.org). All graphs were generated by Excel 2019, the ‘ggplot2’ package in R or Inkscape 1.0. Data normality was first analysed with the Shapiro–Wilk test. Two-tailed Student’s t-test was used for parametric tests whereas for non-parametric tests in Supplementary Figures S3 and S4, the Wilcoxon rank-sum test was applied. For Figure 2, significant differences in gene expression were analysed using a two-way ANOVA with a *post hoc* Tukey’s HSD test.

**Methods S4** **qRT-PCR gene expression studies.**

In three independent trials, eATP-induced gene expression was investigated as described in (Wang et al., 2022) in which 25 - 30 plants (one plate) of either 11-day-old Col-0, *cngc19-1* or *dorn1-3* were pooled together as one biological replicate. Roots were first placed into a 2 mL Eppendorf tube containing half MS liquid medium (pH 5.6, 1 % (w/v) sucrose) for 1.5 h. After that, the solution was replaced by either 0.3 mM Na_2_ATP or 0.6 mM NaCl (prepared in half MS liquid medium, pH 5.6, 1 % (w/v) sucrose, filter sterilised) for eATP treatment or control buffer, respectively. Roots were fully submerged into the solutions for 5 min or 30 min, then dried, excised, and frozen in liquid nitrogen. For Supplementary Figures S3 and S4, total RNA was then extracted with an RNeasy Plant Mini Kit (QIAGEN) then subjected to DNase I treatment (RNase-free DNase kit, QIAGEN). Complementary DNA (cDNA) was synthesised using the QuantiTect Reverse Transcription Kit (QIAGEN). The cDNA for the *cngc2-3* and *cngc2-3,CNGC2::CNGC2* studies was that described by Wang et al. (2022). qRT-PCR was performed in a Rotor-Gene 3000 thermocycler with the Rotor-GeneTM SYBR^®^ Green PCR Kit (QIAGEN). Transcripts were detected using the primers given in Table S1. *AtUBQ10* and *AtTUB4* were used for data normalisation according to the following equation:

$$R_{\mathrm{gene}}=\mathrm{Efficiency}^{-Ct}/\sqrt{(R_{UBQ10})*(R_{TUB4}})$$

In three additional independent trials (results shown in Figure 2) , roots from 11-day-old Col-0, *cngc19-1* and *cngc19-2* were tested. Here, 2 μg of total RNA were obtained using a RNeasy ReliaPrep™ RNA Miniprep System Plant Total RNA kit (Promega) and reverse transcribed using a High-Capacity cDNA Reverse Transcriptase Kit (Applied Biosystems). qRT-PCR was performed with 1 μL of 1:25 diluted cDNA in a total volume of 10 μL using a QuantiNova SYBR Green PCR master mix (Qiagen) on a CFX384 Touch Real-Time PCR detection system (Bio-Rad). Relative quantification of *PEPR1*, *PEPR2*, and *PROPEP1* was determined using the ΔΔ*C*_t_ method (Livak and Schmittgen, 2001) using *AtUBQ10* and *AtTUB4* as reference genes (Vandesompele et al., 2002).

**Table S1** **The primer sequences used in qRT-PCR.**

| **Gene** | **Forward (5' - 3')** | **Reverse (5' – 3')** |
| --- | --- | --- |
| *DORN1*  *(P2K1)* | TGGAGTTTGTCAGGTCCATCG | CTGAGGATCTTCTGCAGGCAA |
| *P2K2* | GGTTTCATGACCATGGAGGCA | ACTTGCACCCCGAATTCCAC |
| *CNGC2* | TCTTCAGGTGGATTGGACTGT | TGGAGGTTTAGTTGCCACCT |
| *CNGC19* | CCTTATTGGAGGTTACGAGCA | ACTACTACTACAAACCAAACCATC |
| *JAZ5* | CAAAGTCAAAGATGTTGCTGACC | GGACTAGAGTTACTCGGATGACT |
| *MPK3* | TGGAGCTTATGGAATCGTTTGCTC | CTGCTTGCTCGATCATCGCTACT |
| *WRKY40* | AGCTTCTGACACTACCCTCGTTG | CACGAGGTTCGACAAGACAGTT |
| *CPK28* | GCCTGAGGAACTTCGAATGCAC | GGTGACGACCTACTTCGTCTGT |
| *PEPR1* | GGCTACATTGCACCAGAAAACG | CGCCACCTGTTTAGAAAGGG |
| *PEPR2* | AAGAAGATGGCTTAATGCTGG | CAGTTGTGCCAGTAACAGTG |
| *PROPEP1* | ACTTCGAAACAGCCGAAGGA | TTTCAATCGAGTCCGGCAGG |
| *TUB4* | AGGGAAACGAAGACAGCAAG | GGTTTCCATCCTAATCGCTCG |
| *UBQ10* | CCGACTACAACATTCAGAAGGA | AAACCTCCACCTCTCAAGACT |

**Video S1 The eATP-induced [Ca^2+^]_cyt_ response in a representative Col-0 root.** GCaMP3-dependent fluorescence was measured for 300 s (images taken every 5 s); 1 µL 0.3 mM eATP treatment was added directly to the root tip at 50 s.

**Video S2 The eATP-induced [Ca^2+^]_cyt_ response in a representative *cngc19-1* root.** GCaMP3-dependent fluorescence was measured for 300 s (images taken every 5 s); 1 µL 0.3 mM eATP treatment was added directly to the root tip at 50 s.

**Fig. S1 Col-0 and *cngc19-1* root [Ca^2+^]_cyt_ response to 0.6 mM NaCl is statistically similar.**

(a) Diagrammatic representation of the *CNGC19* (*Cyclic Nuclotide Gated Channel 19*; AT3G17690) gene including the location of the T-DNA insertion for the *cngc19-1* mutant used here and the *cngc19-2* mutant used by Kundu et al. (2025). Grey boxes denote exons, and the black lines denote introns. GCaMP3 fluorescence (F) was measured for 250 s in Col-0 and *cngc19-1* roots (expressing cytosolic GCaMP3) following a control (0.6 mM NaCl) treatment at 50 s. (b) Intensiometric images (including a brightfield) taken from a single representative Col-0 or *cngc19-1* root across a specified time series. The 0.6 mM NaCl treatment is indicated by a box and images include a 1 mm scale bar. (c) Mean ± SE normalised GCaMP3 fluorescence (ΔF/F_0_) in the full root tip (~2.5 mm) of Col-0 and *cngc19-1* in response to 0.6 mM NaCl. The black arrow indicates application of a 1 µL 0.6 mM NaCl treatment (50 s). The dashed lines specify times of peak ΔF/F_0_ in Col-0 in response to 0.6 mM NaCl (Peak 1, 65 s; Peak 2, 130 s). NaCl-dependent area under the curve (AUC) and peak height analysis are also shown (± SE), with no significant difference (*p*) between Col-0 and *cngc19-1* measured according to a Student’s t*-*test. Peak values shown for *cngc19-1* were determined at the Col-0 peak timepoints to enable comparison. *cngc19-1*’s own Peak 1 height was at 60 s (where *cngc19-1* was 0.3 ± 0.12 and Col-0: 0.24 ±0.1, *p*>0.05). (d) A brightfield image of a root tip showing the two regions of interest (ROI) investigated, ‘A’ and ‘B’. ROI A represents a 150 µm^2^ region found within the first 1 mm of the root apex and ROI B represents a 150 µm^2^ region ~2.5 mm away from the root apex. Mean ± SE normalised GCaMP3 fluorescence (ΔF/F_0_) measured in response to 0.6 mM NaCl in (e) ROI A and (f) ROI B in Col-0 and *cngc19-1* roots. NaCl-dependent area under the curve (A.U.C) and (e) ROI A (Col-0 Peak A1, 75 s; Peak A2, 130 s) and (f) ROI B (Col-0 Peak B1, 70 s; Peak B2, 125 s) ΔF/F_0_ peak height analyses are also included (± SE) for which Peak height values for *cngc19-1* were determined at the peak ΔF/F_0_ Col-0 timepoints. *cngc19-1*’s own ROIB Peak height 1 was recorded at 65 s (where *cngc19-1* was 0.47 ± 0.18 and Col-0: 0.39 ± 0.13, *p*>0.05). The significance value included (*p*) represents the significance of the difference between the values obtained in Col-0 and *cngc19-1* as determined by Student’s *t-*testing. Data were obtained across 6 biological replicates with n = 16 (Col-0) and n = 15 (*cngc19-1*).


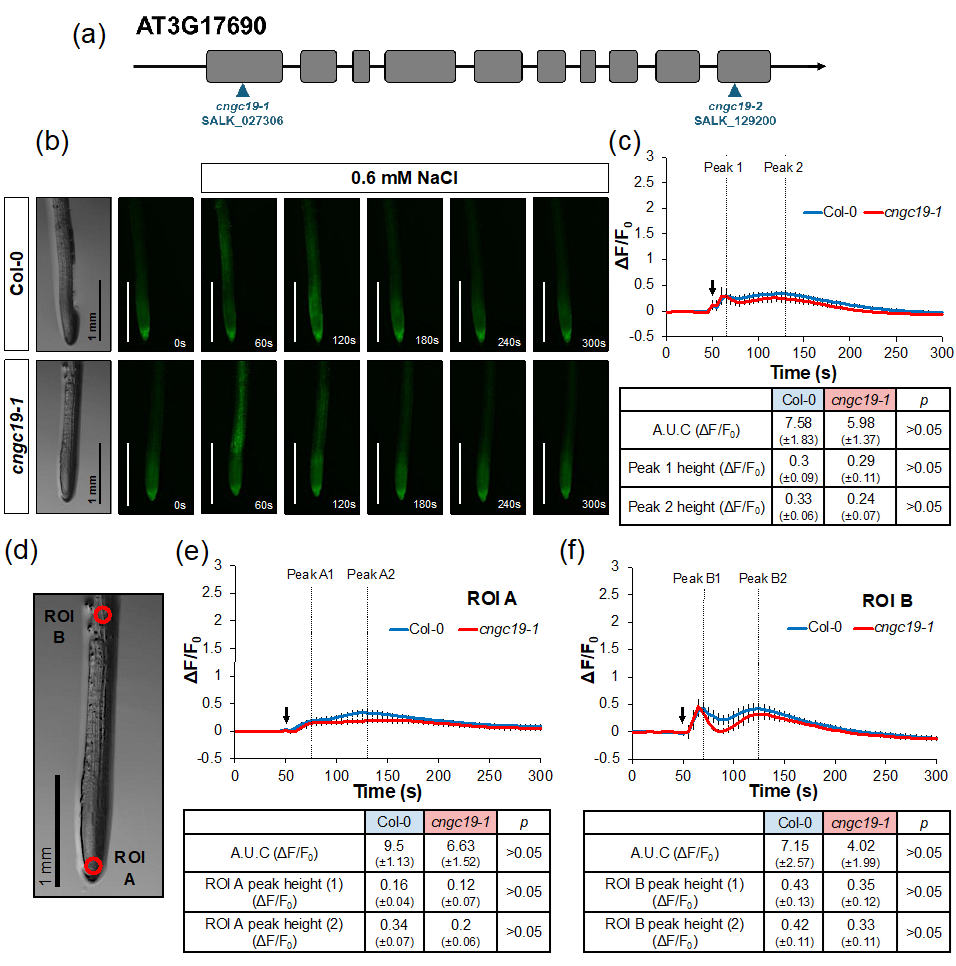


**Video S3 The NaCl-induced [Ca^2+^]_cyt_ response in a representative Col-0 root.** GCaMP3-dependent fluorescence was measured for 300 s (images taken every 5 s); 1 µL 0.6 mM NaCl treatment was added directly to the root tip at 50 s.

**Video S4 The NaCl-induced [Ca^2+^]_cyt_ response in a representative *cngc19-1* root.** GCaMP3-dependent fluorescence was measured for 300 s (images taken every 5 s); 1 µL 0.6 mM NaCl treatment was added directly to the root tip at 50 s.

**Fig. S2 The eATP-induced [Ca^2+^]_cyt_ response is significantly larger than the response to control solution in both Col-0 and *cngc19-1* roots.**

GCaMP3 fluorescence (F) was measured for 250 s in Col-0 and *cngc19-1* (*cyclic nucleotide gated channel19-1* expressing cytosolic GCaMP3) roots following an extracellular ATP (eATP, 0.3 mM; data from Fig. 1) or control (0.6 mM NaCl; data from Fig. S1) treatment at 50 s. Mean ± SE normalised GCaMP3 fluorescence (ΔF/F_0_) in (a and b) the full root tip (~2.5 mm), (c and d) ROI A or (e and f) ROI B in response to 0.3 mM eATP or 0.6 mM NaCl in (a, c and e) Col-0 or (b, d and f) *cngc19-1*. The black arrow indicates application of a 1 µL 0.3 mM eATP or 0.6 mM NaCl treatment (50 s). The ΔF/F_0_ peaks included correspond to the eATP-dependent ΔF/F_0_ peaks from Fig. 1 in (a and b) the full root tip, (c and d) ROI A or (e and f) ROI B. Both NaCl- and eATP-dependent area under the curve (AUC) and peak height analyses are also shown (± SE), with significant differences (*p*) between the response to a 0.3 mM eATP or 0.6 mM NaCl treatment in either Col-0 and *cngc19-1* determined using a Student’s *t-*test.

**Fig. S3** **eATP receptor expression is independent of CNGC19 but eATP-induced *CNGC19* expression requires DORN1/P2K1 and CNGC2.** Whole roots of Col-0, *cngc19-1* (*cyclic nucleotide gated channel19-1*)*, dorn1-3* (*does not respond to nucleotide1-3*), *cngc2-3* (*cyclic nucleotide gated channel2-3*) and *cngc2-3,CNGC2::CNGC2* were treated with 0.3 mM ATP for 5 or 30 min before RNA extraction was carried out and followed by qRT-PCR (quantitative Reverse Transcription-Polymerase Chain Reaction). *UBQ10* (*Poly* *Ubiquitin10*) and *TUB4* (*Tubulin beta chain 4*) were used as housekeeping genes for normalisation. (a) *DORN1/P2K1* (*Does Not Respond to Nucleotide1/P2-type purinoeceptor Kinase 1*) expression in Col-0 and *cngc19-1*. (b) As (a) but *P2K2* (*P2-type purinoeceptor Kinase 2*) expression. (c) *CNGC19* expression in Col-0 and *dorn1-3*. (d) *CNGC19* expression in Col-0, *cngc2-3* and *cngc2-3,CNGC2::CNGC2*. (e) *CNGC2* expression in Col-0 and *cngc19-1*. Data were from the means ± SE of 3 independent trials, with *n* = 4-10 for each genotype and treatment. Data normality was determined by the Shapiro-Wilk test. One-way analysis of variance (two tailed Student’s *t*-test) was used for parametric data, and Wilcoxon rank-sum test was applied for non-parametric data. Asterisks indicate significant differences compared to “No ATP” treatment (*, *p* < 0.05; ***, *p* < 0.001).

**
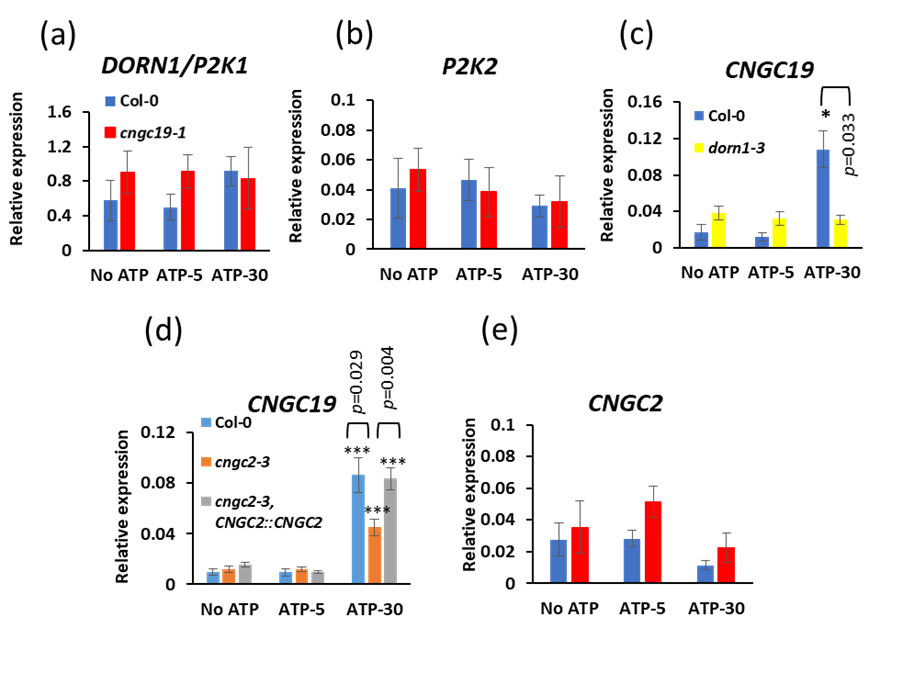
**

**Fig. S4** **CNGC19 is required for eATP-induced *JAZ5, WRKY40* and *MPK3* expression in roots.** Whole roots of Col-0 or *cngc19-1* (*cyclic nucleotide gated channel19-1*) were treated with 0.3 mM ATP for 5 or 30 min before RNA extraction was carried out and followed by qRT-PCR (quantitative Reverse Transcription-Polymerase Chain Reaction). *UBQ10* (*Poly* *Ubiquitin10*) and *TUB4* (*Tubulin beta chain 4*) were used as housekeeping genes for normalisation. (a) *JAZ5* (*Jasmonic Acid ZIM-domain 5*) expression in Col-0 or *cngc19-1*, (b) *WRKY40* (*WRKY DNA-Binding Protein 40*), (c) *MPK3* (*Mitogen-Activated Protein Kinase 3*) and (d) *CPK28* (*Calcium-dependent Protein Kinase 28*). Data were from the means ± SE of 3 independent trials, with *n* = 4-10 for each genotype and treatment. Data normality was determined by the Shapiro-Wilk test. One-way analysis of variance (two tailed Student’s *t*-test) was used for parametric data and Wilcoxon rank-sum test was applied for non-parametric data. Asterisks indicate significant differences compared to “No ATP” treatment (**, *p* < 0.01;***, *p* < 0.001).


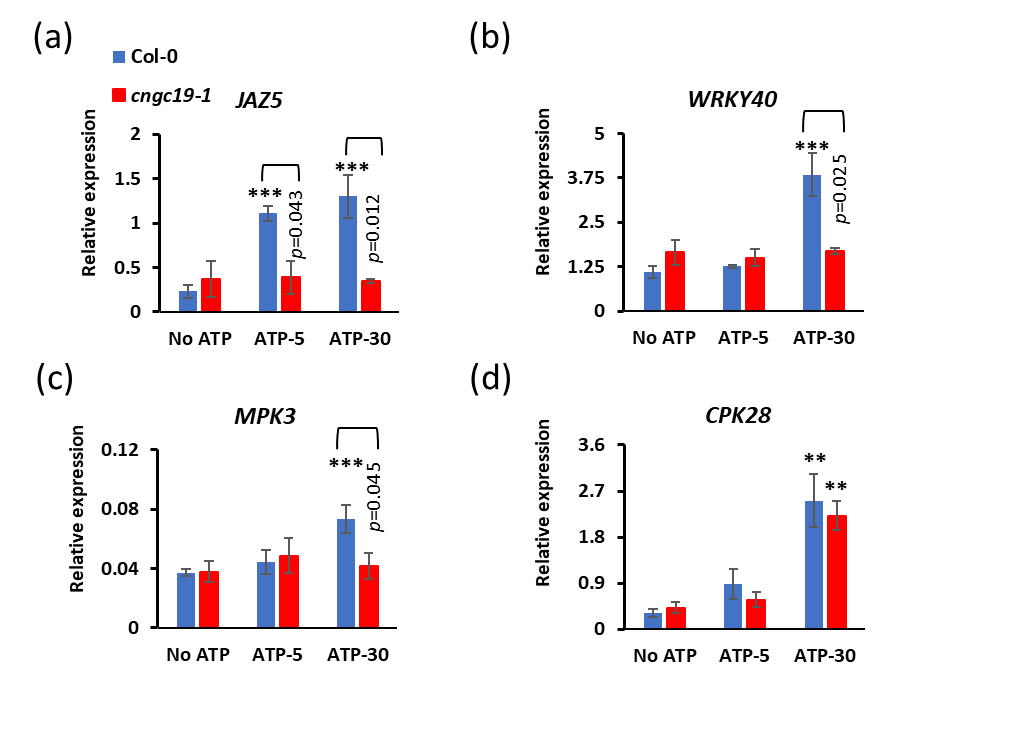


#

**Video S5 The Pep1-induced [Ca^2+^]_cyt_ response in a representative Col-0 root.** GCaMP3-dependent fluorescence was measured for 300 s (images taken every 5 s); 1 µL of 1 μM Pep1 was added directly to the root tip at 50 s. The root shown is the same as presented in Figure S5a.

**Video S6 The Pep1-induced [Ca^2+^]_cyt_ response in a representative *cngc19-1* root.** GCaMP3-dependent fluorescence was measured for 300 s (images taken every 5 s); 1 µL of 1 μM Pep1 was added directly to the root tip at 50 s. The root shown is the same as presented in Figure S5a.

**Video S7 The control-induced [Ca^2+^]_cyt_ response in a representative Col-0 root.** GCaMP3-dependent fluorescence was measured for 300 s (images taken every 5 s); 1 µL of control solution (half MS liquid medium, pH 5.6, 1 % (w/v) sucrose) was added directly to the root tip at 50 s.

**Video S8 The control-induced [Ca^2+^]_cyt_ response in a representative *cngc19-1* root.** GCaMP3-dependent fluorescence was measured for 300 s (images taken every 5 s); 1 µL of control solution (half MS liquid medium, pH 5.6, 1 % (w/v) sucrose) was added directly to the root tip at 50 s.

# **Fig. S5 The root Pep1-induced [Ca^2+^]_cyt_ response is significantly reduced in cngc19-1.**

Pep1-induced fluorescence (F) was measured for 250 s in Col-0 and *cngc19-1* roots (expressing cytosolic GCaMP3) following a 1 µM Pep1 (Peptide1) treatment at 50 s. (a) Intensiometric images (including a brightfield) taken from a single representative Col-0 or *cngc19-1* root across a specified time series. The 1 µM Pep1 treatment is indicated by a box and images include a 1 mm scale bar. (b) Mean ± SE normalised GCaMP3 fluorescence (ΔF/F_0_) in the full root tip (~2.5 mm) in response to 1 µM Pep1 in Col-0 and *cngc19-1*. The black arrow indicates application of 1 µL of 1 µM Pep1 (50 s). Two ΔF/F_0_ peaks (Peak 1, 60 s; Peak 2, 125 s) are shown by dashed lines. eATP-dependent area under the curve (AUC) and peak height analyses are also shown (± SE), with significant differences (*p*) between Col-0 and *cngc19-1* determined by Student’s t-test. (c) Brightfield image of a root tip annotated with the two regions of interest (ROI), ‘A’ and ‘B’. ROI A represents a 150 µm^2^ region found within the first 1 mm of the root apex and ROI B represents a 150 µm^2^ region ~2.5 mm away from the root apex. Mean ± SE normalised GCaMP3 fluorescence (ΔF/F_0_) measured in response to 1 µM Pep1 in (d) ROI A and (e) ROI B in Col-0 and *cngc19-1*. Pep1-induced area under the curve (AUC) and ΔF/F_0_ peak height analyses are also included; (d) ROI A (Peak A1, 70 s; Peak A2, 135 s; Peak A3, 255s) and (e) ROI B (Peak B1, 60 s; Peak B2, 115 s).

The significance value (*p*) for differences between Col-0 and *cngc19-1* were determined by a Student’s t*-*test. Data were obtained across 3 biological replicates with n = 15 (Col-0) and n = 17 (*cngc19-1*). Mean ± SE normalised GCaMP3 fluorescence (ΔF/F_0_) was also measured in response to a respective control treatment (half MS liquid medium, pH 5.6, 1 % (w/v) sucrose) in (f) the full root tip, (g) ROIA and (h) ROIB. AUC and ΔF/F_0_ peak height analyses are also included at the time points used for the Pep1-dependent responses. No significant differences were found between genotypes. The significance value (*p*) for differences between Col-0 and *cngc19-1* were determined by a Student’s *t-*test. Data were obtained across 3 biological replicates with n = 6 (Col-0) and n = 7 (*cngc19-1*).


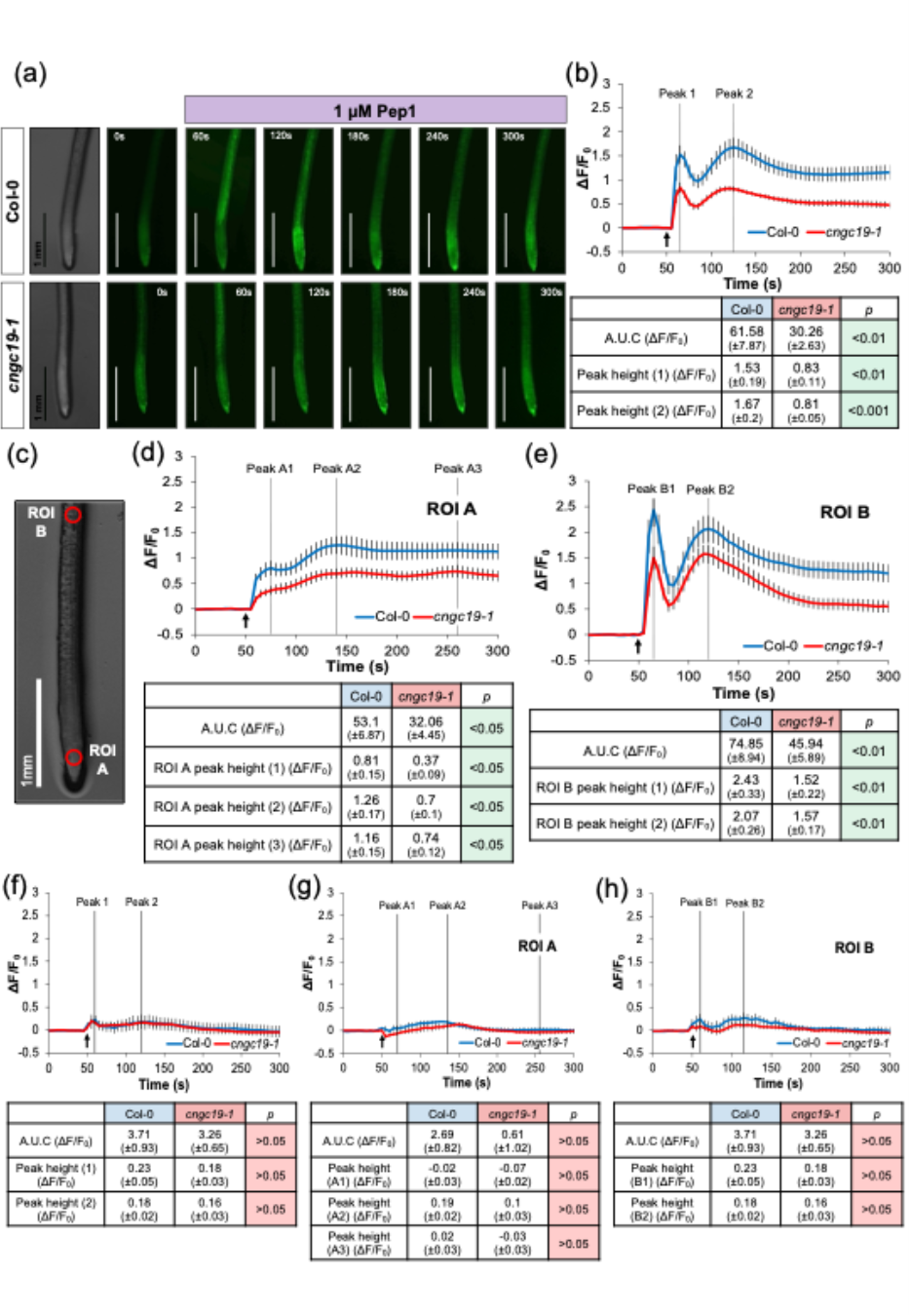


**References**

**Krol E, Mentzel T, Chinchilla D, Boller T, Felix G, Kemmerling B, Postel S, Arents M, Jeworutzki E, Al-Rashied KAS, Becker D, Hedrich H. 2010.** Perception of the *Arabidopsis* danger signal Peptide 1 involves the pattern recognition receptor PEPR1 and its close homologue AtPEPR2. *The Journal of Biological Chemistry* **285**(18): 13471-13479.

**Kugler A, Köhler B, Palme K, Wolff P, Dietrich D. 2009.** Salt-dependent regulation of a CNG channel subfamily in *Arabidopsis.* *BMC Plant Biology* **9**:140.

**Livak KJ, Schmittgen TD. 2001.** Analysis of relative gene expression data using real-time quantitative PCR and the 2-ΔΔ*C*_T_ method. *Methods*, **25**(4), 402–408.

**Matthus E, Wilkins K, Mohammad‐Sidik A, Ning Y, Davies JM. 2022.** Spatial origin of the extracellular ATP‐induced cytosolic calcium signature in *Arabidopsis thaliana* roots: wave formation and variation with phosphate nutrition. *Plant Biology* **24**(5): 863-873.

**Vandesompele J, de Preter K, Pattyn F, Poppe B, van Roy N, de Paepe A, Speleman F. 2002.** Accurate normalization of real-time quantitative RT-PCR data by geometric averaging of multiple internal control genes. *Genome Biology*, **3**(7), 1–12.

**Vincent TR, Avramova M, Canham J, Higgins P, Bilkey N, Mugford ST, Pitino M, Toyota M, Gilroy S, Miller AJ. 2017.** Interplay of plasma membrane and vacuolar ion channels, together with BAK1, elicits rapid cytosolic calcium elevations in *Arabidopsis* during aphid feeding. *The Plant Cell* **29**(6): 1460-1479.

**Wang L, Ning Y, Sun J, Wilkins KA, Matthus E, McNelly RE, Dark A, Rubio L, Moeder W, Yoshioka K, Véry A-A, Stacey G, Leblanc-Fournier N, Legué V, Moulia B, Davies JM. 2022.** *Arabidopsis thaliana* CYCLIC NUCLEOTIDE‐GATED CHANNEL2 mediates extracellular ATP signal transduction in root epidermis. *New Phytologist* **234**(2): 412-421.
